# Biology and Life History of *Balcha indica*, an Ectoparasitoid Attacking the Emerald Ash Borer, *Agrilus planipennis*, in North America

**DOI:** 10.1673/031.011.12701

**Published:** 2011-09-26

**Authors:** Jian J. Duan, Philip B. Taylor, Roger W. Fuester

**Affiliations:** USDA Agricultural Research Station, Beneficial Insects Introduction Research Unit, Newark, DE 19713

**Keywords:** larval parasitoid, life history, wood boring beetles, Buprestidae, Eupelmidae

## Abstract

*Balcha indica* Mani and Kaul (Hymenoptera: Eupelmidae) is a solitary ectoparasitoid attacking larvae, prepupae, and pupae of the emerald ash borer, *Agrilus planipennis* Fairmaire (Hymenoptera: Eupelmidae). Its fecundity, oviposition rate, longevity, and development time were determined in the laboratory under standard rearing conditions (25 ± 2° C, 65 ± 10% relative humidity, and 14:10 L:D). Adults lived a mean of 59 days with a maximum of 117 days. Lifetime adult fecundity averaged 36 eggs with a maximum 94 eggs per female. The egg stage lasted for a maximum of four days with ∼ 50% eggs hatched within two days. The development time of the first instars lasted for a maximum of nine days; 50% of the first instars completed their development (i.e., molted to the next instar) within five days. Instars of the intermediate and final stage larvae (after molting of the first instars occurred) could not be distinguished until they reached the pupal stage, and 50% of those larvae pupated ∼ 62 days after adult oviposition. Under the standard rearing conditions, 50% of *B. indica* took ∼ 83 days to complete the life cycle (from egg to adult emergence) ranging from 47 to 129 days. These results suggest that *B. indica* may not have more than two generations in the mid-Atlantic and Midwest regions of United States, where normal growing seasons—with average temperature above 25° C—are normally less than six months (May–October). Because of the long life span and oviposition period of adults, however, *B. indica* is likely to have overlapping generations.

## Introduction

The emerald ash borer (EAB), *Agrilus planipennis* Fairmaire (Hymenoptera: Eupelmidae), is a relatively new invasive pest that has killed millions of North American ash trees (*Fraxinus* spp.). Destruction by this pest has been pervasive, affecting both managed and natural forests of northeastern and Midwestern states since its discovery in 2002 in Michigan and Ontario, and threatens to kill hundreds of times more across North America (Haack et al. 2002; Kovacs et al. 2010). Currently, EAB has invaded 15 states (Illinois, Indiana, Iowa, Kentucky, Maryland, Michigan, Minnesota, Missouri, New York, Ohio, Pennsylvania, Tennessee, Virginia, West Virginia, and Wisconsin) (Michigan State University 2010) and two Canadian provinces (Ontario and Quebec) (CFIA 2010). Economic cost of potential EAB damage to ash trees has been recently estimated to reach more than $10 billion with possible expansion of infestation to 25 States in the next 10 years (Kovacs et al. 2010). Regulatory efforts to contain the pest's spread via early detection, quarantine, and removal of infested ash trees have had little success (Cappaert et al. 2005; Prasad et al. 2010; Siegert et al. 2010). Moreover, chemical control cannot be used to protect native ashes in forest ecosystems because of prohibitive cost, general impracticality, and potentially negative impacts on the environment (Poland et al. 2006). In contrast, biological control using arthropod natural enemies (primarily parasitoids) may be a cost-effective and environmentally safe alternative. Thus, its potential should be fully explored and rapidly implemented.

Classical biological control efforts against EAB in North America have primarily focused on introduction and releases of parasitoid species collected from northern parts of China (Liu et al. 2003; Yang et al. 2005; Liu et al. 2007; USDA-APHIS 2007; Bauer et al. 2008; Duan et al. 2010). Recently, field surveys in Michigan, Pennsylvania, Ohio, and Ontario also indicated that some existing parasitoids in North America, either indigenous or inadvertently introduced, such as *Atanycolus* spp., *Leuthia astigma*, *Phasgonophora sulcata*, and *Balcha indica* have become associated with EAB and may play a role in its suppression (Bauer et al. 2004; Lyons 2008; Cappaert and McCullough 2009; Duan et al. 2009; Kula et al. 2010). These existing parasitoids may be complementary to the classical biological control agents introduced to suppress EAB in North America.

*Balcha indica* Mani and Kaul (Hymenoptera: Eupelmidae) is one of the 16 world species of *Balcha* that have been recently recognized by Gibson (2005). This eupelmid parasitoid, first collected from unknown wood boring beetles in Virginia in 1995 (Gibson 2005), has since been found in Michigan, Maryland, and Pennsylvania, parasitizing various stages of EAB larvae, prepupae, and/or pupae (Bauer et al. 2004; Gibson 2005; Duan et al. 2009). In some locations, such as western Pennsylvania, *B. indica* appeared to be the most abundant parasitoid attacking EAB populations, resulting in ∼ 4% parasitism (Duan et al. 2009). While its host association with wood-boring beetles is still largely unknown, recent confirmation of its association with the invasive emerald ash borer, *A. planipennis,* raises a new perspective in its potential role in biological control of wood boring pests in North America (Gibson 2005; Duan et al. 2009).

This parasitoid parasitizes various stages of EAB larvae, prepupae, and/or pupae (Bauer et al. 2004; Gibson 2005; Duan et al. 2009). A recent study indicated that *B. indica* reproduces thelytokously (i.e., virgin females reproducing daughters), and may have an advantage in range expansion and establishment by utilizing EAB as its host (Duan et al. 2009). In order to explore the possible use of this parasitoid in an augmentative biological control program against EAB, information on its basic biology and life history traits is needed. The present study investigates the longevity, oviposition rate, and fecundity of adult *B. indica* as well as the developmental time of egg, larvae, and pupae stages on EAB.

## Materials and Methods

### Insects

Adults of *B. indica* originated from larvae and/or pupae parasitizing late instars of EAB collected from Cranberry Township, Pennsylvania. Upon collection from the field, larvae and/or pupae of *B. indica* along with parasitized host (EAB) remains were placed into cells of 12-cell cultural plates lined with wet filter paper, and incubated in an insect rearing room with a controlled ambient temperature (25 ± 2° C), relative humidity (65 ± 10%), and 14:10 L:D until adult wasps emerged. Within 24 hours of emergence as new adults, *B. indica* were transferred into rearing containers made of Crisper boxes (Consolidated Plastics Company, www.consolidatedplastics.com) measuring 17.6 × 12.6 × 10 cm, and ventilated with screened lids and walls. Water was provided to each rearing container hosting individual adult *B. indica* with a dental cotton wick inserted into a 35 ml plastic cup; food in the form of honey was streaked on the screen of the lid and/or wall of the rearing container.

All immature EAB stages (larvae, prepupae, pupae) used in the study were collected from infested ash (*Fraxinus* sp) trees located in Cranberry Township, Pennsylvania, and stored at 2° C for no more than four weeks before their use. Methods for collecting and storing various stages of EAB larvae, prepupae, and/or pupae are described in Duan et al. (2009).

### Adult longevity, oviposition rate, fecundity, and parasitism by *B. indica*

To evaluate adult longevity, oviposition rate, and fecundity of *B. indica,* each female wasp was placed in the rearing container within 24 hours of emergence. Individuals were provided with host larvae (2^nd^ to 4^th^ instars), prepupae, or early stage (white color) pupae inserted into ash twigs (∼ 10 cm long × 1 cm diameter), freshly-cut from green ash (*F. pennsylvanica*) trees using methods described in Duan et al. (2009). Three to 12 host larvae, prepupae, or pupae (inserted into ash twigs) were presented to each female wasp every 2–3 days until the wasp died. The large range in host number used in exposure assays stemmed from the fact that 12 immature hosts were provided to each female in the first week of assays followed by three to six immature hosts in subsequent assays. The reduction of immature EAB hosts from 12 to three or six hosts per female per exposure time was due to the difficulty and costs associated with their collection. It is unlikely this resulted in any negative effects on adult longevity, ovposition rate, fecundity, or parasitism of *B. indica,* because test hosts for each female were replaced frequently (on average twice a week). Additionally, each test wasp did not appear to have used all the test hosts during each exposure period. Bark thickness of twigs was ∼ 1 mm, and likely had no effect on ovipostion rate, fecundity, and parasitism by *B. indica,* which has a ovipositor ranging from 4 to 6 mm long (JJ Duan, unpublished data).

**Table 1.**
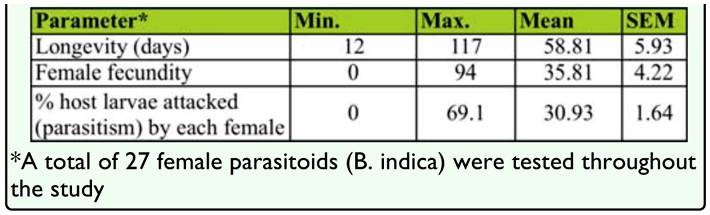
Summary statistics on *Balcha indica* longevity, fecundity and host attack rate.

Throughout the study, a total of 27 female wasps were tested independently, and each female replicate was exposed to a mean of 62 EAB hosts (range: 9–195) and a mean of 15 exposures (range: 3–32). The proportion of host larvae, prepupae, and pupae used in all the exposure assays was 10, 78, and 12%, respectively. Duan et al. (2009) showed that *B. indica* did not exhibit preferences for different immature EAB stages (2^nd^ to 4^th^ instars, prepupae, and pupae) when presented in small ash twigs at approximately equal proportions of each stage. Food and water were also provided *ad libitum* to each wasp within the rearing container during the observation period. While the mortality of adult *B. indica* was observed on a daily basis, oviposition rates and fecundity were determined weekly, based on the number of eggs laid on host larvae by each female wasp. Parasitism was calculated weekly as a proportion of the total number of exposed hosts successfully attacked by each test *B. indica* adult.

### Developmental time of immature stages of *B. indica*

Immediately after being exposed to gravid female parasitoids, ash twigs containing host larvae, prepupae, or pupae were transferred into 200 ml plastic cups secured with screened lids and incubated in an environmental chamber with the same ambient temperature, relative humidity, and photoperiod described above. Developmental stages of immature *B.* *indica* were determined daily over the course of observations. To observe the developmental stages of immature *B. indica,* parasitized hosts were examined under a stereo-microscope by opening up the pre-cut ash bark flap. After parasitoid stages were determined, immature parasitoids and parasitized host remains were rewrapped under the bark flap using Parafilm strips.

**Figure 1.**
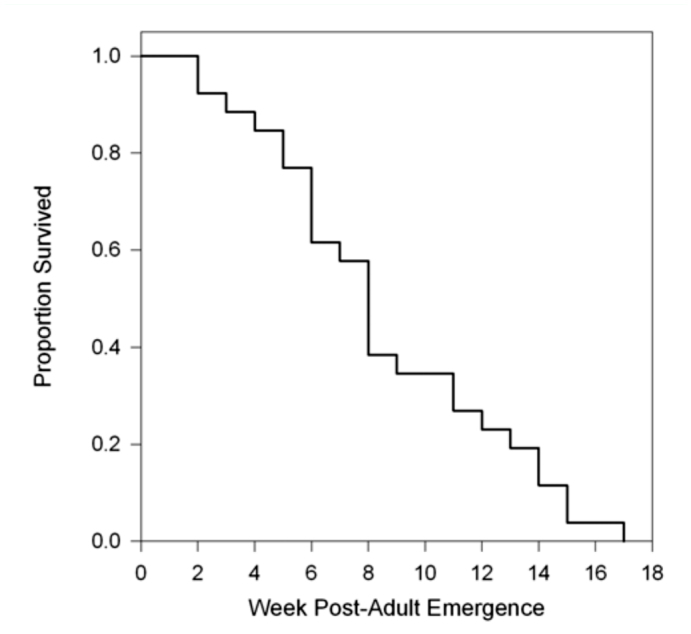
Survivorship of *Balcha indica* ♀♀ over time (N = 26). High quality figures are available online.

### Data Analysis

Data on adult longevity, oviposition rate, fecundity, as well as parasitism rate and developmental times of immature stages of *B. indica* were summarized and analyzed using SAS 9.2.0 (SAS Institute 2004), and presented as means with standard errors (SEM) and 95% confidence intervals (CI).

## Results

### Adult longevity, oviposition rate, fecundity, and parasitism by *B. indica*

**Figure 2.**
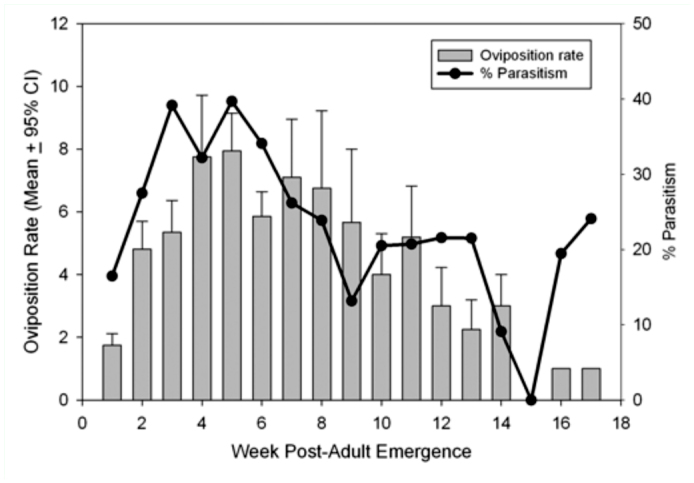
Adult *Balcha indica* oviposition rate (mean number of eggs laid per female ± 95% confidence interval (Cl)) and percent parasitism by *B. indica* reared on immature EAB hosts. High quality figures are available online.

Adults of *B. indica* lived a mean of 58.81 ± 5.93 days (range: 12–117 days) (Table 1), and 50% of the observed adults survived up to eight weeks (Figure 1). Over 96% test females laid more than one egg, and only one female survived 98 days without laying a single egg. Although females started oviposition within ∼ 1 week after emergence, the oviposition rate peaked at four and five weeks after emergence with each female laying a mean of eight eggs per week (Figure 2). Eggs laid through an individual's lifetime measured fecundity of each female *B. indica.* Results showed 35.8 ±4.2 eggs laid per individual, with a maximum of 94 eggs.

Over the entire course of the study, a total of 1613 EAB larvae or pupae were exposed to 27 individuals of *B. indica,* which resulted in 430 successfully parasitized hosts. Although female *B. indica* frequently laid more than one egg on a host, only one parasitoid survived past the first instar and successfully developed to the adult stage. During each bout of exposure (2–6 days), parasitism rate ranged from 0–100% with a mean of 30.9 ± 4.3%. The highest rates of parasitism occurred in females 3–6 weeks old after emergence; a second peak of parasitism occurred with females 16–17 weeks old.

### Developmental time of immature stages of *B. indica*

**Figure 3.**
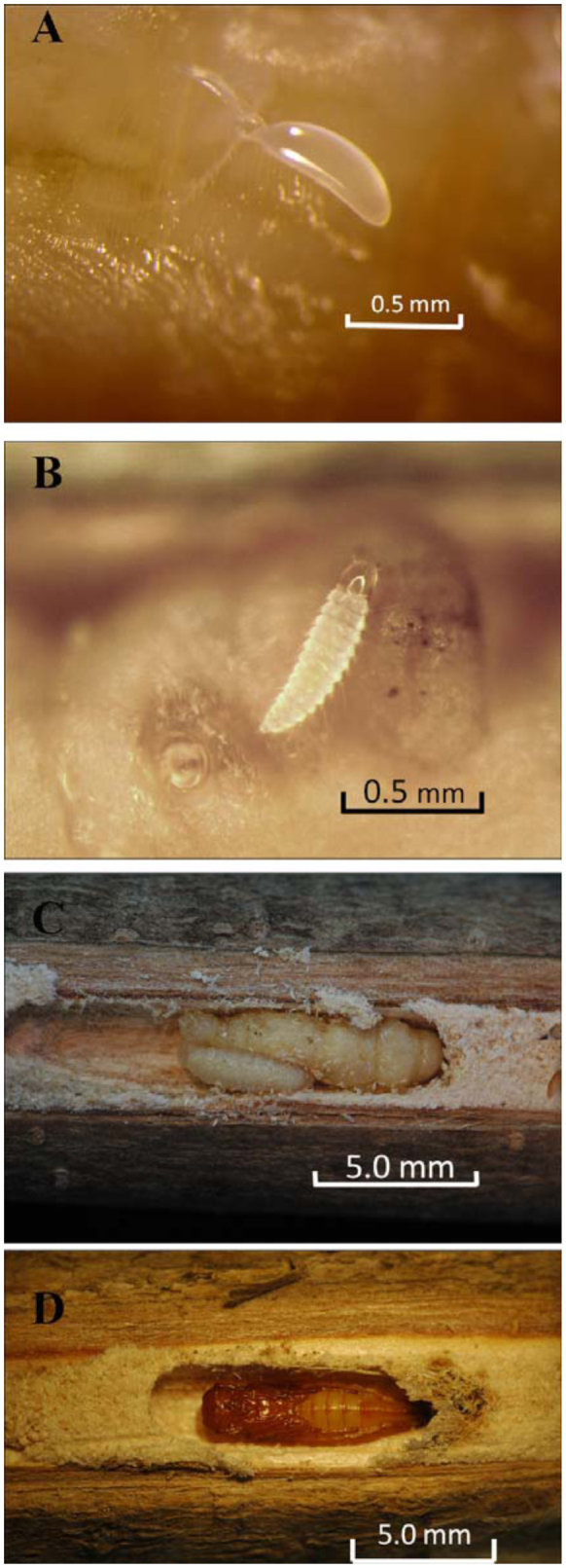
*Balcha indica:* (a) a single egg laid on the surface of an EAB host, (b) 1^st^ instar larva feeding on the EAB pre-pupae, (c) the “maggot-like” intermediate-stage larva parasitizing EAB pupa, and (d) pupa in cell. High quality figures are available online.

Four distinctive immature stages of *B. indica* were recognized during the observation: egg (Figure 3a); first instar (Figure 3b); intermediate/final larval stages (Figure 3c); and pupae (Figure 3d). Females paralyzed the host larvae, prepupae, and/or pupae, and laid one or more eggs on or near the host. Eggs of *B. indica* were often attached to the host via a sticky silk strain. The first instar larva of *B. indica* has a sclerotized head capsule and mandibles, which is followed by the “maggot-like” larval stages, which do not have visible head capsules and mandibles after molting. The instars of those “maggot-like” larval stages could not be distinguished in this study largely because of the lack of apparent characteristics for distinction and a long developmental time. The pupae of *B. indica* have all adult appendages but are not able to move until they molt into adults.

**Figure 4.**
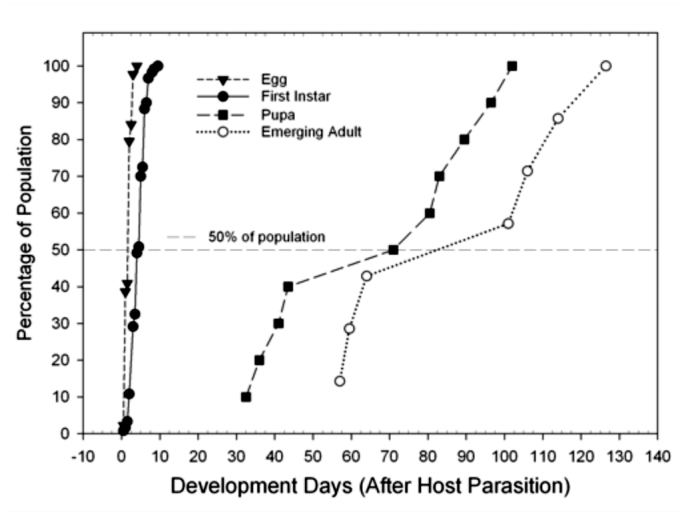
Development rates of immature *Balcha indica* stages (egg through adult) reared on emerald ash borer larvae, prepuape and/or pupae. High quality figures are available online.

The distribution of developmental time, in days, for each immature stage of *B. indica,* from time of oviposition to adult emergence is presented in Figure 4. The egg stage lasted for a maximum of four days, with approximately 50% eggs hatching within two days. The development time of first instars lasted for a maximum of nine days with 50% completing their development within five days. Instars of the “maggot-like” intermediate/final stages of larvae were not distinguished until they reached the pupal stage; 50% pupated 62 days after oviposition. While 50% of the adults emerged approximately 83 days after the hosts were first parasitized, the time of adult emergence ranged from 47 to 129 days after host parasitization.

## Discussion

Findings from this study showed that *B. indica* may take more than three months to complete a single generation (from egg to adult) under standard rearing conditions (25 ± 2° C, 65 ± 10% relative humidity, 14:10 L:D), and had a period of greater than two months for adult longevity and oviposition. Considering a growing season of less than six months (May–October) with daily temperatures averaging ∼ 25° C in the Mid-Atlantic and Midwestern United States, *B. indica* is unlikely to have more than two generations per year in those regions. However, it is very likely that field populations of *B. indica* in those regions will have overlapping generations, resulting from the long (> 2 months) adult longevity and oviposition period.

Recent field surveys conducted in Michigan, Pennsylvania, and Maryland (Bauer et al. 2004; Duan et al. 2009) showed that immature larval stages of *B. indica* were frequently observed on EAB-infested ash trees year-round. In addition, adults of *B. indica* were collected in Michigan, Pennslyvania, and Maryland from various stages of emerald ash borer larvae, prepupae, and pupae in spring, summer, and fall (Duan et al. 2009; JJ Duan, unpublished data). Findings from these field studies along with our laboratory study strongly suggest that *B. indica* may in fact have at least one overlapping generation in the Mid-Atlantic and Midwestern United States, where EAB has recently established. Further field and/or laboratory studies are needed to determine the threshold temperature for normal development and successful overwintering.

Currently, little is known about the biology and life history of *Balcha,* and no study has yet proposed a potential use of this group of parasitoids for biological control. Compared with other groups of hymenopteran parasitoids such as *T. planipennisi* (Ulyshen et al. 2010) and *S. agrili* (Gould et al. 2011) that have been recently introduced from China for biological control of emerald ash borer in the USA, the long life cycle of *B. indica* may not only pose challenges for developing effective rearing methods, but also limit its use as an augmentative biological control agent against the invasive woodborer *A. planipennis.*

On the other hand, lengthy adult longevity and ovipositon periods may be an advantage in establishing an association with *A. planipennis* and other *Agrilus* woodborers in North America, which typically have semivoltine to univoltine generations. In addition, a previous laboratory and field study indicated that *B. indica* reproduce through thelytokous parthenogenesis (i.e., virgin females producing daughters) and is capable of attacking a broad range of immature stages of emerald ash borer from second instar to pupae (Duan et al. 2009). Thus, it is likely that this parasitoid could be complementary to current classical biological control programs against EAB in North America, which thus far have focused primarily on the introduction of exotic larval and egg parasitoids from China. However, the potential impact of competitive interaction by *B. indica,* along with newly introduced biological control agents *T. planipennisi* and *S. agrili* that attack similar immature stages of EAB larvae (e.g., Ulyshen et al. 2010), should be further investigated.

## References

[bibr01] Bauer LS, Liu H-P, Haack RA, Miller DL, Patrice TR, Mastro V, Reardon R (2004). Natural enemies of emerald ash borer in southeastern Michigan.. *Proceedings of the Emerald Ash Borer Research and Technology Meeting..*.

[bibr02] Bauer LS, Liu H, Miller DL, Gould J (2008). Developing a Classical Biological Control Program for *Agrilus planipennis* (Coleoptera: Buprestidae), an Invasive Ash Pest in North America.. *Newsletter of the Michigan Entomological Society*.

[bibr03] Canadian Food Inspection Agency. (2010). *Emerald Ash Borer - Agrilus planipennis.*.

[bibr04] Cappaert D, McCullough DG (2009). Occurrence and seasonal abundance of *Atanycolus cappaerti* (Hymenoptera: Braconidae) a native parasitoid of emerald ash borer, *Agrilus planipennis* (Coleoptera: Buprestidae).. *Great Lakes Entomologist*.

[bibr05] Cappaert D, McCullough DG, Poland TM, Siegert NW (2005). Emerald ash borer in North America: A research and regulatory challenge.. *American Entomologist*.

[bibr06] Duan JJ, Fuester RW, Wildonger J, Taylor PB, Barth S, Spichiger SE (2009). Parasitoids attacking the emerald ash borer (Coleoptera: Buprestidae) in Western Pennsylvania.. *Florida Entomologist*.

[bibr07] Duan JJ, Ulyshen MD, Bauer LS, Gould JR, van Driesche R (2010). Measuring the impact of biotic factors on populations of immature emerald ash borers (Coleoptera: Buprestidae).. *Environmental Entomology*.

[bibr08] Gibson GAP (2005). The world species of *Balcha* Walker (Hymenoptera: Chalcidoidea: Eupelmidae), parasitoids of wood-boring beetles.. Zootaxa 1033..

[bibr09] Gould JR, Ayer T, Fraser I (2011). Effect of rearing conditions on reproduction by *Spathius agrili* (Hymenoptera: Braconidae), a parasitoid of the emerald ash borer (Coleoptera: Buprestidae).. *Journal of Economic Entomology* (In press).

[bibr10] Haack RA, Jendek E, Liu H, Marchant KR, Petrice TR, Poland TM, Ye H (2002). The emerald ash borer: a new exotic pest in North America.. *Newsletter of the Michigan Entomological Society*.

[bibr11] Kovacs KF, Haight RG, McCullough DG, Mercader RJ, Siegert NW, Liebhold AM (2010). Cost of potential emerald ash borer damage in U.S. communities, 2009–2019.. *Ecological Economics*.

[bibr12] Kula RR, Knight KS, Rebbeck J, Bauer LS, Cappaert DL, Gandhi KJ (2010). *Leluthia astigma* (Ashmead) (Hymenoptera: Braconidae: Doryctinae) as a parasitoid of *Agrilus planimennis* Fairmarie (Coleoptera: Buprestidae: Agrilinae), with an assessment of host associations for nearctic species of *Leluthia* Cameron.. *Proceedings of Entomological Society of Washington*.

[bibr13] Liu H, Bauer LS, Gao R, Zhao T, Petrice TR, Haack RA (2003). Exploratory survey for the emerald ash borer, *Agrilus planipennis* (Coleoptera: Buprestidae), and its natural enemies in China.. *Great Lakes Entomologist*.

[bibr14] Liu H, Bauer LS, Miller DL, Zhao T, Gao R, Song L, Luan Q, Jin R, Gao C (2007). Seasonal abundance of *Agrilus planipennis* (Coleoptera: Buprestidae) and its natural enemies *Oobius agrili* (Hymenoptera: Encyrtidae) and *Tetrastichus planipennisi* (Hymenoptera: Eulophidae) in China.. *Biological Control: Theory and Application in Pest Management*.

[bibr15] Lyons B (2008). Emerald ash borer: It's here to stay, let's learn how to manage it.. *Forest Health and Biodiversity Newsletter*.

[bibr16] Michigan State University. (2010). Emerald Ash Borer Information for Homeowners..

[bibr17] Prasad AM, Iverson LR, Peters MP, Bossenbroek JM, Matthews SN, Sydnor TD, Schwartz MW (2010). Modeling the invasive emerald ash borer risk of spread using a spatially explicit cellular model.. *Landscape Ecology*.

[bibr18] Poland TM, McCullough DG (2006). Emerald ash borer: invasion of the urban forest and the threat to North America's ash resource.. *Journal of Forestry*.

[bibr19] SAS Institute. (2004). *SAS/STAT User's Guide*, Version 9.1..

[bibr20] Siegert NW, McCullough DG, Williams DW, Fraser I, Poland TM, Pierce SJ (2010). Dispersal of *Agrilus planipennis* (Coleoptera: Buprestidae) from discrete epicenters in two outlier sites.. *Environmental Entomology*.

[bibr21] USDA-APHIS. (2007). The proposed release of three parasitoids for the biological control of the emerald ash borer (*Agrilus planipennis*) in the continental United States: environmental assessment.. *Federal Register*.

[bibr22] Ulyshen MD, Duan JJ, Bauer LS, Ivich F (2010). Suitability and accessibility of immature *Agrilus planipennis* (Coleoptera: Buprestidae) stages to *Tetrastichus planipennisi* (Hymenoptera: Eulophidae).. *Journal of Economic Entomology*.

[bibr23] Yang Z-Q, Strazanac JS, Marsh PM, Van Achterberg C, Choi W-Y (2005). First recorded parasitoid from China of *Agrilus planipennis* : A new species of *Spathius* (Hymenoptera: Braconidae: Doryctinae).. *Annals of the Entomological Society of America*.

